# Emerging Executive Functioning and Motor Development in Infants at High and Low Risk for Autism Spectrum Disorder

**DOI:** 10.3389/fpsyg.2016.01016

**Published:** 2016-07-05

**Authors:** Tanya St. John, Annette M. Estes, Stephen R. Dager, Penelope Kostopoulos, Jason J. Wolff, Juhi Pandey, Jed T. Elison, Sarah J. Paterson, Robert T. Schultz, Kelly Botteron, Heather Hazlett, Joseph Piven

**Affiliations:** ^1^Speech and Hearing Sciences, University of WashingtonSeattle, WA, USA; ^2^UW Autism Center, Center on Human Development and Disability, University of WashingtonSeattle, WA, USA; ^3^Department of Radiology, University of WashingtonSeattle, WA, USA; ^4^McConnell Brain Imaging Centre, Montreal Neurological InstituteMontreal, CA, USA; ^5^Department of Educational Psychology, University of MinnesotaMinneapolis, MN, USA; ^6^Department of Pediatrics, The Center for Autism Research, Children's Hospital of Philadelphia, University of PennsylvaniaPhiladelphia, PA, USA; ^7^Institute of Child Development, University of MinnesotaMinneapolis, MN, USA; ^8^Department of Psychiatry, Washington University School of MedicineSaint Louis, MO, USA; ^9^Carolina Institute for Developmental DisabilitiesChapel Hill, NC, USA; ^10^Department of Psychiatry, University of North CarolinaChapel Hill, NC

**Keywords:** executive functioning, autism, high-risk, motor skills, working memory, inhibition

## Abstract

Existing evidence suggests executive functioning (EF) deficits may be present in children with autism spectrum disorder (ASD) by 3 years of age. It is less clear when, prior to 3 years, EF deficits may emerge and how EF unfold over time. The contribution of motor skill difficulties to poorer EF in children with ASD has not been systematically studied. We investigated the developmental trajectory of EF in infants at high and low familial risk for ASD (HR and LR) and the potential associations between motor skills, diagnostic group, and EF performance. Participants included 186 HR and 76 LR infants. EF (A-not-B), motor skills (Fine and Gross Motor), and cognitive ability were directly assessed at 12 months and 24 months of age. Participants were directly evaluated for ASD at 24 months using DSM-IV-TR criteria and categorized as HR-ASD, HR-Negative, and LR-Negative. HR-ASD and HR-Negative siblings demonstrated less improvement in EF over time compared to the LR-Negative group. Motor skills were associated with group and EF performance at 12 months. No group differences were found at 12 months, but at 24 months, the HR-ASD and HR-Negative groups performed worse than the LR-Negative group overall after controlling for visual reception and maternal education. On reversal trials, the HR-ASD group performed worse than the LR-Negative group. Motor skills were associated with group and EF performance on reversal trials at 24 months. Findings suggest that HR siblings demonstrate altered EF development and that motor skills may play an important role in this process.

## Introduction

Autism spectrum disorder (ASD) is a neurodevelopmental disorder characterized by deficits in social communication and repetitive behaviors (DSM-5, American Psychiatric Association, 2013). Core behavioral features of ASD appear to emerge within the first 2 years of life (see Brian et al., 2008; Estes et al., 2015). Atypicalities associated with ASD include executive functioning (EF) deficits and motor impairments. The age at which EF deficits and motor impairments emerge in children who go on to develop ASD and their developmental trajectories are not well understood (Hughes et al., 1994; Hughes, 1996; Happé et al., 2006; Solomon et al., 2008; Corbett et al., 2009; Bhat et al., 2011). Although not considered defining features of ASD, deficits in these domains may be markers for later developmental problems and precede the unfolding of ASD symptoms in the first years of life.

EF refers to a set of cognitive functions that include attention, inhibition of behavior, working memory, cognitive flexibility, planning, and problem solving (Diamond, 2013). EF emerges progressively and increases in complexity with age during typical development (Best and Miller, 2010; Chevalier, 2015). The timing of the emergence of these functions and their developmental progression remains unclear (Diamond, 2002; Best and Miller, 2010; Jones et al., 2014). The ability to inhibit prepotent responses may be among the first functions to emerge (Chevalier, 2015). By 12 months of age infants can effectively inhibit their actions and engage their working memory to find a hidden toy (Diamond and Goldman-Rakic, 1989). There is even evidence of emerging working memory in 6-month old infants (Gilmore and Johnson, 1995). Cognitive flexibility may be the latest EF to emerge (Davidson et al., 2006; Garon et al., 2008). Research is needed to understand whether differences in EF emerge as part of early ASD symptoms and whether EF may be part of a developmental cascade that contributes to later deficits in individuals with ASD.

A broad range of EF impairments have been found in individuals with ASD including impairments in planning, working memory, inhibiting behavior, and cognitive flexibility (Hughes et al., 1994; Hughes, 1996; Hill, 2004a; Happé et al., 2006; Solomon et al., 2008; Corbett et al., 2009). Verbal and Non-verbal IQ is related to performance of EF tasks measuring inhibition, working memory, and cognitive flexibility in children with ASD (Griffith et al., 1999; Dawson et al., 2002; Yerys et al., 2007; Faja et al., 2016). Evidence suggests that deficits in EF may be present in children with ASD by 3 years of age (McEvoy et al., 1993; Adrien et al., 2001; Holmboe et al., 2008), although not all studies support this finding (Griffith et al., 1999; Dawson et al., 2002; Yerys et al., 2007). Griffith et al. (1999), in one of the few longitudinal studies of EF in ASD, found that preschool-aged children with ASD committed the same number of perseverative errors on a spatial reversal task over time while a comparison group of children with developmental delay showed a trend reduction in errors on this task as they grew older. Cross-sectional studies further support the idea of altered EF trajectories of working memory and response inhibition in children with ASD. For example, Luna et al. (2007) found evidence that typically developing individuals improved their performance on a computerized task tapping working memory as age increased whereas the ASD group did not. Similarly, Solomon et al. (2008) found that individuals with ASD did not improve their performance on a task assessing working memory and response inhibition as age increased but rather slightly worsened in their performance over time. All studies to date have focused on EF deficits after symptoms of ASD have manifested and relatively little is known about EF capabilities prior to and during the unfolding of ASD symptoms.

EF and motor development may be interrelated because of underlying neurobiology, as suggested by evidence from behavior-based tasks (Diamond, 2000). Functional neuroimaging studies have found co-activation in the dorsolateral prefrontal cortex and cerebellum on EF tasks such as the Wisconsin card sorting test (Berman et al., 1995) as well as on several working memory tasks not requiring motor-based responses (Awh et al., 1996; Desmond et al., 1997; Hautzel et al., 2009; Durisko and Fiez, 2010). Studies have also found that there are neuronal pathways linking the dorsolateral prefrontal cortex and neocerebellum (see Diamond, 2000 for further discussion). In typically developing infants, the experience of self-locomotion, such as walking, is associated with success on the A-not-B, a task measuring inhibition of prepotent responses and working memory (see Smith et al., 1999). Various motor functions, such as reaching and standing, may also influence performance on the A-not-B (Smith et al., 1999). Furthermore, gross and fine motor skills have been associated with working memory, verbal fluency, and cognitive flexibility (Wassenberg et al., 2005; Livesey et al., 2006; Piek et al., 2008). Older children (12–13 years) with ASD have shown difficulty with tasks requiring simultaneous goal-directed and motor behavior (Hughes, 1996). Interestingly, interventions that improve motor skills in children with ASD may improve certain aspects of EF, such as working memory (Hilton et al., 2014). However, the contribution of early motor skill difficulties to the emergence of EF problems in children who later develop ASD has not been systematically studied.

In this study, we investigated the longitudinal patterns of EF performance in infant siblings at high and low risk for ASD. Siblings of children with ASD are at higher risk for developing ASD compared with siblings of children who do not have an older sibling with ASD. The recurrence rate of risk for ASD in HR siblings is estimated to be between 10 and 18 % (Constantino et al., 2010; Ozonoff et al., 2011) while the population prevalence rate is 1 in 68^1^. High-risk (HR) infants, with older siblings with ASD and low-risk infants (LR), with typically developing older sibling were divided into three outcome groups based on ASD diagnosis at 24 months; HR-ASD, (HR infants who developed ASD) HR-negative (HR infants who did not develop ASD), and LR-Negative (LR infants who did not develop ASD). It was hypothesized that the HR-ASD group would demonstrate less improvement in EF over time and lower EF at 12 and 24 months than the HR-Negative and LR-Negative groups. We further hypothesized that motor skills would be associated with EF performance and group.

## Methods

### Participants

Infants at high-familial risk for ASD due to an older sibling with ASD (HR; *n* = 186) and low familial risk for ASD, with typically developing older sibling and no family history of ASD (LR; *n* = 76) were included in this study. HR infants had an older sibling who met criteria for ASD on the Social Communication Questionnaire (SCQ; Rutter et al., 2003) and Autism Diagnostic Interview, Revised (ADI-R; Lord et al., 1994) and had an ASD diagnosis, confirmed by medical records. LR infants had typically developing older siblings who did not meet cut off scores for ASD on the SCQ or Family Interview for Genetics Studies (FIGS; Maxwell, 1992) and had no first-degree relative with ASD or intellectual disability. All participants were screened and excluded based on the following: (1) birth weight < 2000 g and/or gestational age < 36 weeks or significant perinatal adversity and/or exposure *in utero* to neurotoxins, (2) medical/neurological conditions affecting growth, development, or cognition (e.g., seizure disorder) or significant sensory impairments (e.g., vision or hearing loss), (3) genetic conditions or syndromes, (4) adopted children or half siblings, (5) twins, (6) first-degree relative with psychosis, schizophrenia, or bipolar disorder (FIGS), (7) contraindication for MRI and, (8) predominant home language other than English.

### Procedures

The sample included participants who provided valid A-not-B data at either the 12- or 24-month time point. Participants were recruited through research participant lists, flyers, brochures, email blasts, and community clinics at four clinical sites (Children's Hospital of Philadelphia, University of Washington, University of North Carolina, and Washington University). Following eligibility screening, participants were assessed at 6, 12, and 24 months of age. Written informed consent, approved by each site's Human Subjects Review Board, was obtained for all families.

Cognitive, social development, and EF performance were assessed by a licensed clinical psychologist, doctoral student in clinical psychology, school psychologist, or masters-level psychometrist under supervision of a licensed clinical psychologist or psychiatrist. At 24 months, all participants were assessed using the Mullen Scales of Early Learning (MSEL; Mullen, 1995), the Autism Diagnostic Observation Schedule, Second Edition (ADOS-2; Lord et al., 2012) and ADI-R by research-reliable examiners. Each participant was assigned a clinical best estimate diagnosis made by two clinicians according to the DSM-IV-TR criteria to determine whether the child met the criteria for Autistic Disorder, Pervasive Developmental Disorder-Not Otherwise Specified, or neither. There were 30 high-risk infants meeting DSM-IV criteria for Autistic Disorder or PDD-NOS (HR-ASD), 138 high-risk infants *not* meeting DSM-IV criteria for Autistic Disorder or PDD-NOS (HR-Negative), and 67 low-risk infants *not* meeting DSM-IV-TR criteria for Autistic Disorder or PDD-NOS (LR-Negative).

Table 1 presents demographic and descriptive information including age, gender, race, and maternal education. Groups did not differ in age, race, or sex at either the 12- or 24-month assessment. However there were group differences in maternal education at 24 months, $\chi_{\left(2,\text{N}=172\right)}^{2}$ = 7.37, *p* = 0.025 but not 12 months, $\chi_{\left(2,\text{N}=173\right)}^{2}=$ 5.64, *p* = 0.059. Groups differed significantly on MSEL Fine Motor skills at 12 months, *F*_(2, 171)_ = 3.48, *p* = 0.033, and at 24 months, *F*_(2, 171)_ = 9.56, *p* < 0.001. Groups also differed significantly on MSEL Gross Motor skills at 12 and 24 months *F*_(2, 171)_ = 3.36, *p* = 0.037, *F*_(2, 171)_ = 8.90, *p* < 0.001, respectively. On the Visual Reception scale of the MSEL, groups differed significantly at 24 months but not at 12 months, *F*_(2, 171)_ = 4. 21, *p* = 0.016, *F*_(2, 171)_ = 2.89, *p* = 0.058, respectively. Floor and ceiling effects were assessed across groups by examining score ranges. Few participants in any group fell at either the floor or ceiling values. Twenty-four participants with A-not-B had missing diagnostic outcome data and 3 LR siblings were diagnosed with ASD and, therefore, were not included in the analysis. In addition, 40 HR-Negative, 13 HR-ASD, and 22 LR-Negative infants at 12 months (χ^2^ = 0.83, *p* = 0.662) and 20 HR-Negative, 9 HR-ASD, and 5 LR-Negative infants at 24 months (χ^2^ = 7.11, *p* = 0.029) did not provide valid A-not-B data due to training failure, completion of too few trails (< 10), or administration errors. Further details about the characteristics of this cohort can be found in Estes et al. (2015).

**Table 1:** **Study sample characteristics**.

	**HR-ASD**	**HR-Negative**	**LR-Negative**	***p*^a^**
**12 MONTHS**				
*n*	23	101	50	
Age (months)	12.49 (0.52)	12.52 (0.67)	12.55 (0.79)	0.934
Race (%)				
White	78.3	85.0	87.8	0.576
Non-white	21.7	15.0	12.2	
Sex (% male)	73.9	56.4	58.0	0.300
Maternal education (%)				0.059
No College degree	47.8	30.7	20.4	
College degree	52.2	69.3	79.6	
MSEL Visual Reception ^b^	50.30 (9.53)	54.14 (9.27)	55.74 (8.07)	0.058
MSEL Fine Motor ^b^	54.35 (9.00)	58.18 (8.58)	60.02 (8.23)	0.033
MSEL Gross Motor ^b^	44.78 (13.18)	47.77 (11.87)	52.02 (12.19)	0.037
**24 MONTHS**				
*n*	19	106	49	
Age (months)	24.26 (0.90)	24.63 (0.90)	24.63 (1.05)	0.270
Race (%)				0.210
White	73.7	87.6	89.4	
Non-white	26.3	12.4	10.6	
Sex (% male)	73.7	59.4	49.0	
Maternal education (%)				0.025
No College degree	42.1	33.3	14.6	
College degree	57.9	66.7	85.4	
MSEL Visual Reception ^b^	49.58 (6.96)	52.93 (10.21)	56.80 (10.73)	0.016
MSEL Fine Motor ^b^	45.89 (9.47)	49.75 (8.77)	55.14 (8.79)	0.000
MSEL Gross Motor ^b^	42.26 (8.63)	49.82 (9.15)	52.04 (7.29)	0.000

a *Omnibus ANOVA (Age, MSEL) and Chi-Square (race, maternal education, sex)*.

b *T-scores*.

## Measures

### Executive function

EF was assessed with the A-not-B task (Piaget, 1954) at 12 and 24 months. The A-not-B has been used as a measure of response inhibition and working memory in children as young as 6 months of age (Diamond, 1985; Diamond and Goldman-Rakic, 1989). The implementation of this task has varied across prior studies. In the current study the infant watched as a toy was hidden to the left or right of midline and was encouraged to find the toy after a delay of 5 s. Once the infant found the hidden toy on two consecutive trials, the side of hiding was reversed. The delay was increased to 12 s if the infant successfully completed two reversal trials. A maximum of 24 trials and 4 reversal trials were administered. Performance was measured by two criteria: (1) proportion of total correct reaches by total trials (working memory) and (2) the proportion of total correct reaches on reversal trials by total reversals trials (inhibition).

#### Cognitive ability and motor skills

The Mullen Scales of Early Learning (MSEL; Mullen, 1995) is a standardized, normed, developmental assessment for children birth through 68 months. The MSEL yields 5 subscales (Receptive Language, Expressive Language, Visual Reception, Fine Motor, and Gross Motor) and the Early Learning Composite (ELC), an overall index of cognitive ability. The Gross and Fine Motor, and Visual Reception subscale T-score were used in this study. The Visual Reception scale was used as a proxy for overall cognitive ability because the ELC and Non-verbal developmental quotient include the Fine Motor scale, which was used as an independent variable in this study.

### ASD symptoms

The Autism Diagnostic Observation Schedule, second edition (ADOS-2; Lord et al., 2012) is a semi-structured play assessment of communication, social interaction, play skills, and restricted interests/repetitive behavior. Module 1 was administered to all children at 24 months. Empirically derived algorithm scores, based on the severity and number of ASD symptoms demonstrated during the ADOS assessment, yield three classifications, Autism, Autism Spectrum, and Non-Spectrum.

The Autism Diagnostic Interview-Revised (ADI-R; Lord et al., 1994) is a semi-structured parent interview that assesses symptoms of ASD. The ADI-R was administered at 24 months to all parents of HR infants and all LR infants with ASD-related clinical concerns. The ADOS and ADI-R contributed to a clinical best estimate ASD diagnosis.

### Statistical analysis

EF development from ages 12 to 24 months was analyzed using generalized estimating equations (GEE) fit for a binomial distribution and exchangeable correlation matrix using SPSS version 19.0. Dependent variables included the proportion of total correct reaches by total trials (measuring working memory) and total correct reaches on reversal trials by total reversal trials (measuring response inhibition) on the A-not-B. Model predictors included diagnostic group (HR-ASD, HR-Negative, and LR-Negative), maternal education, and the MSEL Visual Reception subscale. Cross-sectional group differences in EF were tested employing logistic regression at 12 and 24 months (see Marcovitch and Zelazo, 1999) using the same dependent variables and model predictors. Separate regression models were run to determine if motor skills were associated with group and EF at 12 and 24 months using the same dependent variables and model predictors, with the addition of the MSEL Fine and Gross Motor subscales.

## Results

### EF development

#### Working memory

**Figure 1:**
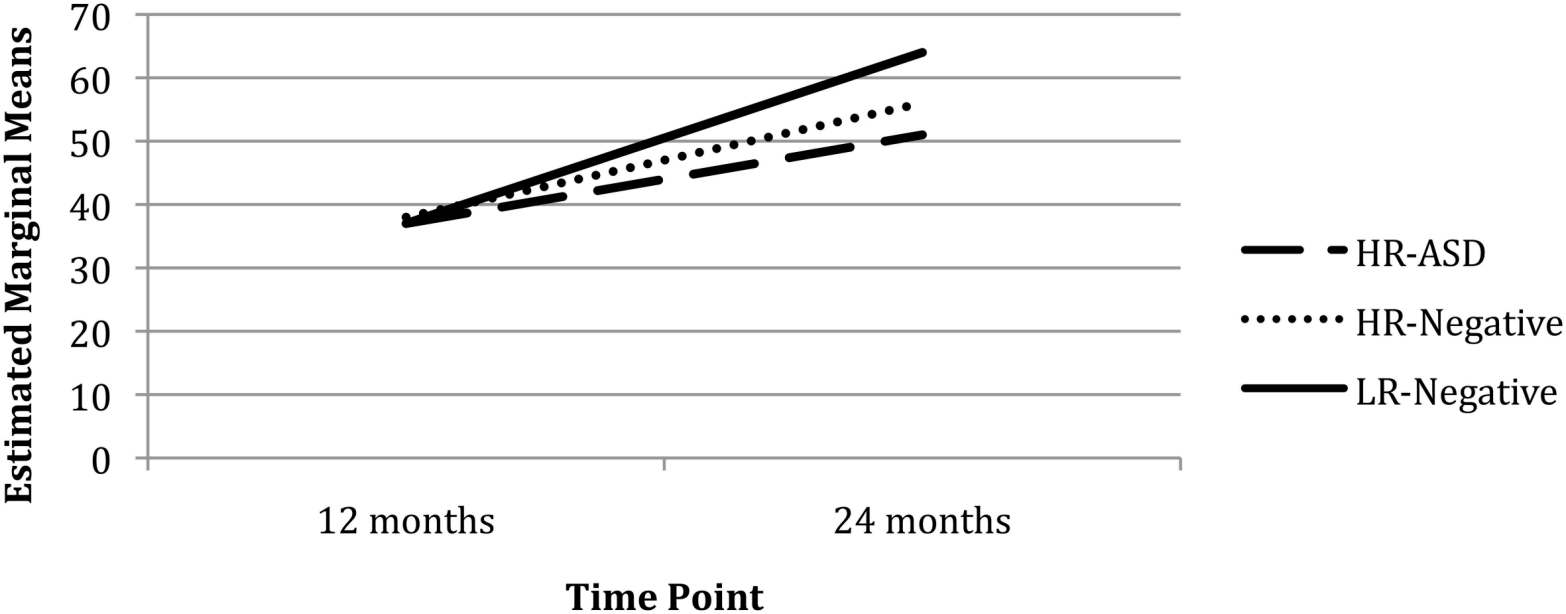
**Total correct reaches by total trials over time**.

There was a significant main effect of Time (χ^2^ = 67.73, *p* < 0.001) but not Group (χ^2^ = 3.91, *p* = 0.141) on working memory (proportion of total correct reaches by total trials) after controlling for Visual Reception and maternal education. There was a significant Group x Time interaction for working memory with the LR-Negative group demonstrating improved performance from 12 to 24 months (χ^2^ = 48.60, *p* < 0.001) and the HR-ASD (χ^2^ = 3.98, *p* = 0.046) and HR-Negative (χ^2^ = 4.62, *p* = 0.032) groups demonstrating less improvement than LR-Negative group (see Figure 1).

#### Response inhibition

**Figure 2:**
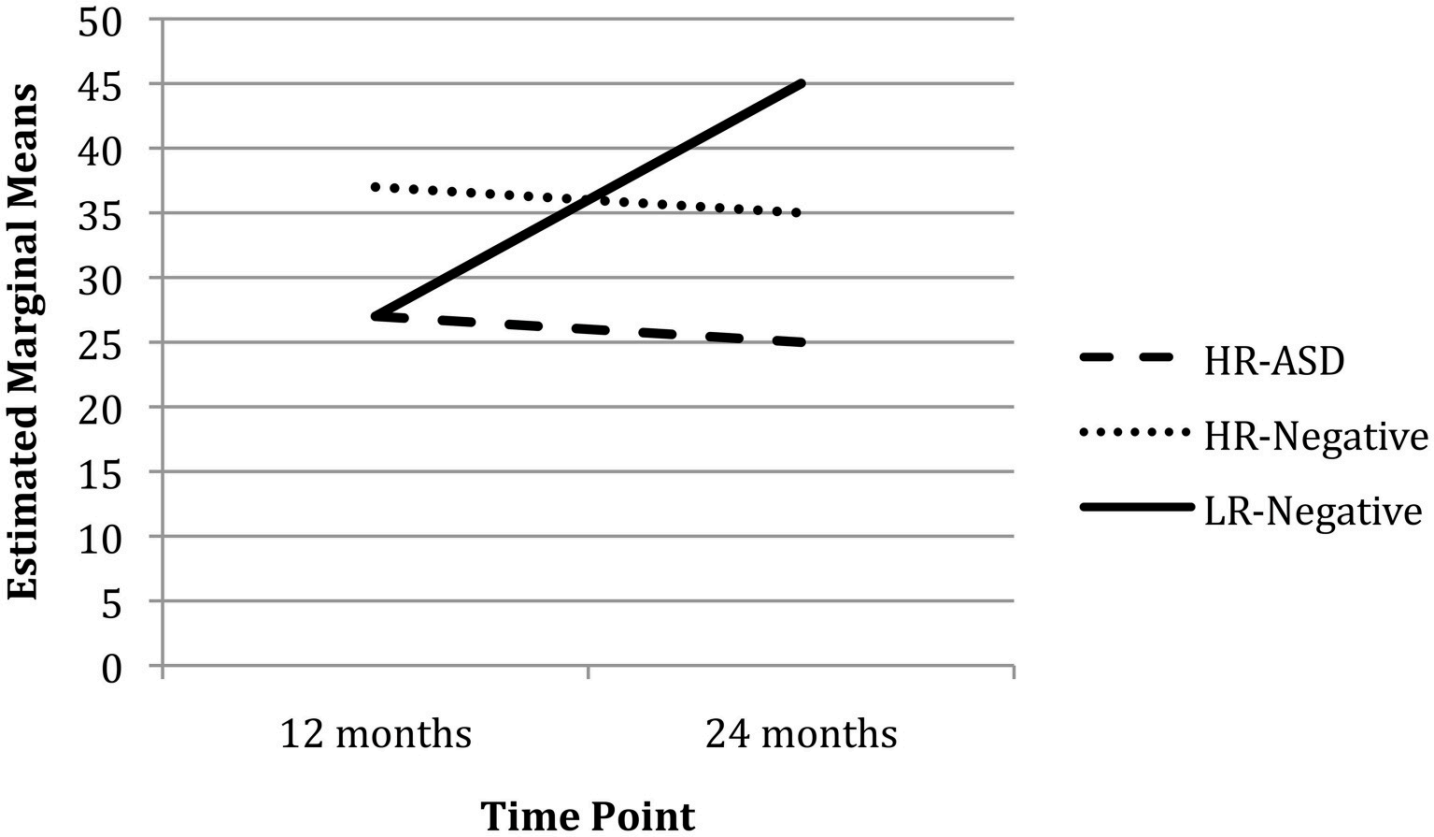
**Total correct reaches on reversal trials by total reversal trials over time**.

There was a significant main effect of Time (χ^2^ = 6.79, *p* = 0.009) but not Group (χ^2^ = 2.15, *p* = 0.342) on response inhibition (proportion of total correct reaches on reversal trials by total reversal trials) after controlling for Visual Reception and maternal education. The Group x Time interaction was significant for response inhibition with the LR-Negative group improving performance from 12 to 24 months and the HR-Negative group slightly worsening performance over time (χ^2^ = 5.48, *p* = 0.019; see Figure 2). The HR-ASD group also worsened over time but no interaction effect was detected (χ^2^ = 2.18, *p* = 0.140). *Post-hoc* analysis, adding the Fine and Gross Motor subscales as covariates, did not change the pattern or significance of the results reported above.

#### Group differences in EF and motor at 12 months

##### Group differences in working memory

There was no significant main effect of group on working memory at 12 months after controlling for maternal education and Visual Reception (χ^2^ = 0.39, *p* = 0.822; see Model 1, Table 2).

**Table 2:** **Summary of model fit for working memory and fine motor at 12 months**.

**Covariate**	**Model 1 95% CI**					**Model 2 95% CI**					**Model 3 95% CI**				
	**B**	**SE**	**LL**	**UL**	***P***	**B**	**SE**	**LL**	**UL**	***p***	**B**	**SE**	**LL**	**UL**	***p***
No College Degree^a^	0.07	0.08	−0.10	0.24	0.406	0.05	0.09	−0.12	0.22	0.545	0.05	0.09	−0.12	0.22	0.576
Visual Reception	0.00	0.00	−0.01	0.01	0.853	0.00	0.00	−0.01	0.01	0.422	0.00	0.00	−0.01	0.01	0.545
HR−ASDb	0.00	0.13	−0.26	0.25	0.976	−0.06	0.13	−0.32	0.19	0.634	1.35	0.82	−0.27	2.96	0.102
HR-Negative ^b^	0.05	0.09	−0.12	0.22	0.598	0.02	0.09	−0.16	0.19	0.854	0.77	0.62	−0.45	1.99	0.214
Fine Motor						−0.01	0.00	−0.02	−0.01	0.001	0.00	0.01	−0.02	0.02	0.745
HR-ASD^*^Fine Motor ^c^											−0.03	0.01	−0.05	0.00	0.086
HR-Negative^*^Fine Motor ^c^											−0.01	0.01	−0.03	0.01	0.223

a *Reference group = college degree*.

b *Reference group = LR-Negative*.

c Reference group = LR-Negative ^*^ Fine Motor.

##### Fine motor and working memory

Lower Fine Motor scores were associated with better working memory at 12 months (χ^2^ = 10.52, *p* ≤ 0.001, see Model 2, Table 2). The Group × Fine Motor interaction at 12 months was not significant (χ^2^ = 3.09, *p* = 0.213; see Model 3, Table 2).

##### Gross motor and working memory

There was no main effect of Gross Motor on working memory at 12 months (χ^2^ = 2.72, *p* = 0.099, see Model 2, Table 3). The Group × Gross Motor interaction at 12 months was not significant (χ^2^ = 0.13, *p* = 0.938; see Model 3, Table 3).

**Table 3:** **Summary of model fit for working memory and gross motor at 12 months**.

**Covariate**	**Model 1 95% CI**					**Model 2 95% CI**					**Model 3 95% CI**				
	**B**	**SE**	**LL**	**UL**	***p***	**B**	**SE**	**LL**	**UL**	***p***	**B**	**SE**	**LL**	**UL**	***p***
No College Degree^a^	0.07	0.08	−0.10	0.24	0.406	0.07	0.09	−0.10	0.23	0.444	0.06	0.09	−0.10	0.23	0.451
Visual Reception	0.00	0.00	−0.01	0.01	0.853	0.00	0.00	−0.01	0.01	0.625	0.00	0.00	−0.01	0.01	0.624
HR-ASD ^b^	0.00	0.13	−0.26	0.25	0.976	0.02	0.13	−0.23	0.27	0.880	0.15	0.53	−0.90	1.19	0.780
HR-Negative^b^	0.05	0.09	−0.12	0.22	0.598	0.07	0.09	−0.11	0.24	0.456	0.20	0.40	−0.57	0.98	0.611
Gross Motor						0.01	0.00	0.00	0.01	0.099	0.01	0.01	−0.01	0.02	0.264
HR-ASD^*^ Gross Motor ^c^											0.00	0.01	−0.02	0.02	0.811
HR-Negative^*^ Gross Motor ^c^											0.00	0.01	−0.02	0.01	0.726

a *Reference group = college degree*.

b *Reference group = LR-Negative*.

c Reference group = LR-Negative ^*^ Gross Motor.

##### Group differences in response inhibition

There was no significant main effect of group on response inhibition at 12 months after controlling for maternal education and Visual Reception (χ^2^ = 3.43, *p* = 0.180, see Model 1, Table 4).

**Table 4:** **Summary of model fit for response inhibition and fine motor at 12 months**.

**Covariate**	**Model 1 95% CI**					**Model 2 95% CI**					**Model 3 95% CI**				
	**B**	**SE**	**LL**	**UL**	***p***	**B**	**SE**	**LL**	**UL**	***p***	**B**	**SE**	**LL**	**UL**	***p***
No College^a^	0.13	0.26	−0.39	0.64	0.621	0.09	0.26	−0.43	0.61	0.731	0.13	0.27	−0.39	0.66	0.616
Visual Reception	0.02	0.01	−0.01	0.05	0.115	0.03	0.01	0.00	0.06	0.040	0.03	0.01	0.00	0.06	0.062
HR-ASD^b^	−0.01	0.40	−0.80	0.78	0.972	−0.18	0.41	−0.99	0.63	0.660	7.07	3.17	0.86	13.28	0.026
HR-Negative^b^	0.43	0.27	−0.11	0.97	0.116	0.37	0.28	−0.18	0.91	0.185	3.12	2.11	−1.03	7.26	0.140
Fine Motor						−0.03	0.01	−0.06	0.00	0.046	0.02	0.03	−0.04	0.08	0.571
HR-ASD ^*^ Fine Motor ^c^											−0.13	0.06	−0.25	−0.02	0.024
HR-Negative^*^ Fine Motor^c^											−0.05	0.03	−0.11	0.02	0.191

a *Reference group = college degree*.

b *Reference group = LR-Negative*.

c Reference group = LR-Negative ^*^ Fine Motor.

##### Fine motor and response inhibition

Lower Fine Motor scores were associated with better response inhibition at 12 months (χ^2^ = 4.00, *p* = 0.046, see Model 2, Table 4). The Group × Fine Motor interaction at 12 months was significant. The HR-ASD group demonstrated a negative association between Fine Motor scores and response inhibition and the LR-Negative group demonstrated a positive relationship (χ^2^ = 5.10, *p* = 0.024; see Model 3, Table 4).

##### Gross motor and response inhibition

There was no significant main effect of Gross Motor scores on response inhibition at 12 months (χ^2^ = 1.27, *p* = 0.258, see Model 2, Table 5). The Group × Gross Motor interaction was not significant (χ^2^ = 1.39, *p* = 0.498; see Model 3, Table 5).

**Table 5:** **Summary of model fit for response inhibition and gross motor at 12 months**.

**Covariate**	**Model 1 95% CI**					**Model 2 95% CI**					**Model 3 95% CI**				
	**B**	**SE**	**LL**	**UL**	***p***	**B**	**SE**	**LL**	**UL**	***p***	**B**	**SE**	**LL**	**UL**	***p***
No College^a^	0.13	0.26	−0.39	0.64	0.621	0.16	0.26	−0.36	0.68	0.552	0.17	0.27	−0.35	0.69	0.524
Visual Reception	0.02	0.01	−0.01	0.05	0.115	0.03	0.01	0.00	0.05	0.074	0.03	0.01	0.00	0.06	0.063
HR-ASD^b^	−0.01	0.40	−0.80	0.78	0.972	−0.08	0.41	−0.88	0.72	0.843	−0.53	1.71	−3.88	2.82	0.756
HR-Negative^b^	0.43	0.27	−0.11	0.97	0.116	0.38	0.28	−0.17	0.92	0.176	−1.01	1.32	−3.59	1.57	0.443
Gross Motor						−0.01	0.01	−0.03	0.01	0.258	−0.03	0.02	−0.08	0.01	0.169
HR-ASD^*^ Gross Motor^c^											0.01	0.03	−0.06	0.08	0.833
HR-Negative^*^ Gross Motor ^c^											0.03	0.03	−0.02	0.08	0.280

a *Reference group = college degree*.

b *Reference group = LR-Negative*.

c Reference group = LR-Negative ^*^ Gross Motor.

#### Group differences in EF and motor at 24 months

##### Group differences in working memory

There was a significant main effect of group on working memory at 24 months after controlling for Visual Reception and maternal education with the HR-ASD and HR-Negative groups performing worse than the LR-Negative group (χ^2^ = 18.83, *p* < 0.001; see Model 1, Table 6). Estimated marginal means and standard errors were generated from this model. Bonferroni corrected pair-wise comparisons further indicated that the HR-ASD and HR-Negative groups did not differ significantly from each other (Omnibus χ^2^ = 19.67, *p* ≤ 0.001).

**Table 6:** **Summary of model fit for working memory and fine motor at 24 months**.

**Covariate**	**Model 1 95% CI**					**Model 2 95% CI**					**Model 3 95% CI**				
	**B**	**SE**	**LL**	**UL**	***p***	**B**	**SE**	**LL**	**UL**	***p***	**B**	**SE**	**LL**	**UL**	***p***
No College Degree^a^	0.32	0.08	0.16	0.49	0.000	0.33	0.08	0.16	0.49	0.000	0.33	0.08	0.17	0.50	0.000
Visual Reception	0.02	0.00	0.01	0.02	0.000	0.01	0.00	0.00	0.02	0.011	0.01	0.00	0.00	0.02	0.011
HR-ASD^b^	−0.52	0.14	−0.79	−0.25	0.000	−0.43	0.14	−0.71	−0.16	0.002	−0.36	0.76	−1.84	1.13	0.639
HR-Negative^b^	−0.34	0.09	−0.51	−0.16	0.000	−0.28	0.09	−0.46	−0.11	0.002	0.15	0.54	−0.90	1.21	0.776
Fine Motor						0.01	0.00	0.01	0.02	0.003	0.02	0.01	0.00	0.04	0.029
HR-ASD^*^ Fine Motor^c^											0.00	0.02	−0.03	0.03	0.965
HR-Negative^*^ Fine Motor ^c^											−0.01	0.01	−0.03	0.01	0.408

a *Reference group = college degree*.

b *Reference group = LR-Negative*.

c Reference group = LR-Negative ^*^ Fine Motor.

##### Fine motor and working memory

Higher Fine Motor scores were associated with better working memory (χ^2^ = 9.06, *p* = 0.003, see Model 2, Table 6). The Group × Fine Motor interaction was not significant (χ^2^ = 0.84, *p* = 0.656). In the interaction model there was no longer a significant main effect for group (see Model 3, Table 6).

##### Gross motor and working memory

There was no main effect for Gross Motor on working memory (χ^2^ = 2.73, *p* = 0.098; see Model 2, Table 7). The Group × Gross Motor interaction was also not significant (χ^2^ = 5.73, *p* = 0.057). However, in the interaction model, there was a significant main effect of Gross Motor skills on working memory (χ^2^ = 3.95, *p* = 0.047) and there was no longer a significant main effect for group (see Model 3, Table 7).

**Table 7:** **Summary of model fit for working memory and gross motor at 24 months**.

**Covariate**	**Model 1 95% CI**					**Model 2 95% CI**					**Model 3 95% CI**				
	**B**	**SE**	**LL**	**UL**	***p***	**B**	**SE**	**LL**	**UL**	***p***	**B**	**SE**	**LL**	**UL**	***p***
No College Degree ^a^	0.32	0.08	0.16	0.49	0.000	0.32	0.08	0.15	0.482	0.000	0.3	0.1	0.1	0.5	0.000
Visual Reception	0.02	0.00	0.01	0.02	0.000	0.02	0.00	0.01	0.022	0.000	0.0	0.0	0.0	0.0	0.000
HR-ASD ^b^	−0.52	0.14	−0.79	−0.25	0.000	−0.47	0.14	−0.75	−0.19	0.001	0.7	0.6	−0.5	1.9	0.244
HR-Negative ^b^	−0.34	0.09	−0.51	−0.16	0.000	−0.32	0.09	−0.50	−0.147	0.000	−0.7	0.8	−2.3	0.9	0.403
Gross Motor						0.01	0.00	0.00	0.015	0.098	0.02	0.01	0.00	0.04	0.047
HR-ASD ^*^ Gross Motor ^c^											0.01	0.02	−0.03	0.04	0.648
HR-Negative ^*^ Gross Motor ^c^											−0.02	0.01	−0.04	0.00	0.087

a *Reference group = college degree*.

b *Reference group = LR-Negative*.

c Reference group = LR-Negative ^*^ Gross Motor.

##### Group differences in response inhibition

There was a significant main effect of group on response inhibition at 24 months after controlling for Visual Reception and maternal education with the HR-ASD group and HR-Negative group performing worse than the LR-Negative group (χ^2^ = 7.39, *p* = 0.025; see Model 1, Table 7). Bonferonni corrected pair-wise comparisons indicated that only the HR-ASD and LR-Negative groups differed (Omnibus χ^2^ = 7.85, *p* = 0.020).

##### Fine motor and response inhibition

There was no main effect of Fine Motor scores on response inhibition at 24 months (χ^2^ = 3.56, *p* = 0.059; see Model 2, Table 8). The Group × Fine Motor interaction was also not significant (χ^2^ = 3.18, *p* = 0.204; see Model 3, Table 8). In the interaction model, Fine Motor significantly predicted response inhibition (χ^2^ = 5.22, *p* = 0.022) with the main effect for group no longer reaching significance.

**Table 8:** **Summary of model fit for response inhibition on reversal trials and fine motor at 24 months**.

**Covariate**	**Model 1 95% CI**					**Model 2 95% CI**					**Model 3 95% CI**				
	**B**	**SE**	**LL**	**UL**	***p***	**B**	**SE**	**LL**	**UL**	***p***	**B**	**SE**	**LL**	**UL**	***p***
No College^a^	−0.05	0.20	−0.44	0.34	0.815	−0.03	0.20	−0.42	0.37	0.901	0.02	0.20	−0.38	0.42	0.927
Visual Reception	0.01	0.01	−0.01	0.03	0.227	0.00	0.01	−0.02	0.02	0.734	0.00	0.01	−0.02	0.02	0.727
HR-ASD^b^	−0.88	0.36	−1.59	−0.17	0.016	−0.78	0.37	−1.50	−0.06	0.034	−0.21	1.85	−3.84	3.42	0.909
HR-Negative^b^	−0.40	0.19	−0.78	−0.02	0.038	−0.33	0.20	−0.72	0.06	0.098	1.71	1.23	−0.69	4.11	0.162
Fine Motor						0.02	0.01	0.00	0.04	0.059	0.04	0.02	0.01	0.08	0.022
HR-ASD^*^ Fine Motor^c^											−0.01	0.04	−0.08	0.06	0.816
HR-Negative^*^ Fine Motor ^c^											−0.04	0.02	−0.08	0.01	0.090

a *Reference group = college degree*.

b *Reference group = LR-Negative*.

c Reference group = LR-Negative ^*^ Fine Motor.

##### Gross motor and response inhibition

Higher Gross Motor scores were associated with better response inhibition at 24 months (χ^2^ = 4.90, *p* = 0.027; see Model 2, Table 9). The Group by Gross Motor interaction was significant at 24 months with the HR-Negative and LR-Negative groups showing a positive relationship between Gross Motor and performance on reversal trials but with the HR-Negative group demonstrating lower scores overall (χ^2^ = 3.89, *p* = 0.049; see Model 3, Table 9).

**Table 9:** **Summary of model fit for response inhibition and gross motor at 24 months**.

**Covariate**	**Model 1 95% CI**					**Model 2 95% CI**					**Model 3 95% CI**				
	**B**	**SE**	**LL**	**UL**	***p***	**B**	**SE**	**LL**	**UL**	***p***	**B**	**SE**	**LL**	**UL**	***p***
No College^a^	−0.05	0.20	−0.44	0.34	0.815	−0.05	0.20	−0.44	0.35	0.820	−0.02	0.20	−0.42	0.37	0.917
Visual Reception	0.01	0.01	−0.01	0.03	0.227	0.01	0.01	−0.01	0.03	0.389	0.01	0.01	−0.01	0.03	0.286
HR-ASD^b^	−0.88	0.36	−1.59	−0.17	0.016	−0.74	0.37	−1.46	−0.01	0.047	1.76	2.07	−2.31	5.83	0.396
HR-Negative^b^	−0.40	0.19	−0.78	−0.02	0.038	−0.36	0.20	−0.74	0.03	0.069	2.21	1.31	−0.37	4.78	0.093
Gross Motor						0.02	0.01	0.00	0.04	0.027	0.06	0.02	0.02	0.10	0.005
HR-ASD^*^ Gross Motor^c^											−0.05	0.04	−0.13	0.04	0.261
HR-Negative^*^ Gross Motor ^c^											−0.05	0.02	−0.10	0.00	0.049

a *Reference group = college degree*.

b *Reference group = LR-Negative*.

c Reference group = LR-Negative ^*^ Gross Motor.

## Discussion

The current study investigated the early emergence of EF in children at high and low risk for ASD, prior to the onset of the disorder. HR infants who later developed ASD and HR infants who did not develop ASD showed slower growth in working memory from 12 to 24 months of age than LR infants without ASD. The LR-Negative group showed improved response inhibition from 12 to 24 months while the HR-ASD and HR-Negative groups showed little to no improvement in inhibition. At 12 months no group differences in working memory or inhibition were evident. Differences emerged by 24 months with the HR-ASD and HR-Negative groups demonstrating worse working memory and response inhibition than the LR-Negative group. These findings are consistent with prior research suggesting altered trajectories of EF in children with ASD (Griffith et al., 1999; Luna et al., 2007; Solomon et al., 2008). These findings are also consistent with emerging evidence that EF differences may be present in HR-Negative siblings (Hill, 2004b; Holmboe et al., 2010; Warren et al., 2012). This study is unique in providing the earliest evidence to date of EF differences in children with ASD, suggesting that these deficits may emerge in the second year of life, around the same time that the core symptoms of ASD are also emerging and consolidating.

In the current study, evidence was also found that motor skills are associated with EF performance and diagnostic group. At 12 months, worse fine motor skills were associated with better working memory and response inhibition. Interestingly, when looking specifically at differences across groups, the HR-ASD group demonstrated an inverse relationship between fine motor skills and response inhibition, with better fine motor skills related to worse response inhibition. The LR-Negative group demonstrated the expected relationship with better response inhibition related to better fine motor skills. Gross motor skills, however, were not related to EF at 12 months of age. At 24 months, better fine and gross motor skills were associated with better working memory and response inhibition. Fine motor skills were not associated with group and EF but worse gross motor skills were associated with worse response inhibition. These findings are consistent with prior research reporting an association between motor skills and the A-not-B (see Smith et al., 1999) and add to the growing evidence suggesting a relationship between EF and motor skills in both typically developing children and children with ASD (Hughes, 1996; Diamond, 2000; Hilton et al., 2014).

Interestingly, HR-Negative siblings demonstrated differences in working memory compared to the LR-Negative group but not compared to the HR-ASD group, suggesting that EF differences may be a manifestation of genetic liability for ASD. Having atypical EF development could reflect a more general genetic vulnerability for developmental problems among HR siblings who do not develop full clinical symptoms of ASD. HR-Negative siblings may demonstrate a range of developmental problems such as lower developmental functioning, language delay, social difficulties, and greater internalizing problems (Landa et al., 2012; Georgiades et al., 2013; Messinger et al., 2013; Pisula and Ziegart-Sadowska, 2015). This is the first study of which we are aware to demonstrate that EF may be impaired in HR siblings who do not develop ASD. Some problems in HR-Negative siblings, such as language impairments and social-emotional problems, may be related to EF deficits, as suggested by the literature in non-ASD populations (Riggs et al., 2006; Henry et al., 2012). Future studies are needed to determine if EF and developmental problems are associated in HR-Negative siblings.

Another notable finding is that HR-ASD siblings had more difficulty inhibiting behavior than LR-Negative siblings at 24 months. The HR-Negative siblings did not differ significantly from either group and performed better than HR-ASD siblings but worse than LR-Negative siblings. It is possible that the working memory demands of the A-not-B were lower than the inhibitory demands which may indicate that children with ASD have more difficulty on tasks that put greater demand on the their EFs but that this may be less of a difficulty for HR siblings that do not go on the develop ASD. Underlying neural differences in HR-ASD siblings could account for differences in response inhibition. For example, decreased activation in the regions associated with inhibition on a behavioral inhibition task in children with ASD has been found (Kana et al., 2007). Another interesting finding was that EF differences in HR-ASD siblings were, in some cases, opposite of what was found in LR-Negative siblings. Again, this could be related to some altered, underlying brain-based process or be the effect of the type of EF that is being engaged. Finally, the impact of motor development on EF performance was noted earlier than overt differences in working memory and response inhibition were observed. This pattern of early emerging, motor-based differences has been found in other studies of HR-ASD siblings (Bolton et al., 2012; Flanagan et al., 2012; Estes et al., 2015). However, the current study, to our knowledge, is the first to tie these differences to EF.

Contrary to expectations, differences in working memory and response inhibition at 12 months of age was not demonstrated. It may be that differences in working memory and response inhibition do not exist as this age. However, Holmboe et al. (2010) found poorer inhibition on a computer-based EF task in 9–10 month old HR infant siblings compared to LR infant siblings, which suggests that EF deficits may exist at young ages but may be hard to detect with motor-based EF tasks. In addition, there may be some specific, developmentally related reason accounting for infant's performance at 12 months of age, such as stage of self-locomotion (e.g., walking) or preference for bi-manual reaching while learning to walk (which is an “incorrect” response on the A-not-B; Smith et al., 1999; Corbetta and Bojczyk, 2002; Karasik et al., 2011). It is also possible that the infants in our study were already fatigued due to unmeasured factors (e.g., time of day, prior cognitive testing, hunger etc.). Moreover, it was unexpected that poorer Fine Motor performance on the Mullen would be associated with better EF performance at 12 months. No prior literature, as far as we are aware, has described a similar finding. Future studies are needed to clarify whether this relationship is due to random variability in our sample, measure fidelity, or reflects a unique relationship between ASD and motor development. If these findings are replicated in independent sample, a possible explanation could be that poorer Fine Motor skills and better A-not-B performance are different sides of the same coin, both related to early ASD symptoms. Specifically, ASD is often related to a preference for objects. Thus, it is possible that the A-not-B, an object-based task, may be inherently rewarding for an infant with emerging ASD symptoms. ASD is also related to difficulties with imitation and motor development, which may lead to lower scores on some of the Fine Motor items. Thus, subtle difficulties with imitation or fine motor skills may impact Mullen performance but not necessarily A-not-B performance and subtle preferences for objects may increase A-not-B performance in the younger ages. However, these considerations are not addressable with the current data set and future studies are needed to replicate and extend this work.

Early findings of EF deficits in school-age children and adults with ASD lead to the theory that EF deficits may be a primary deficit explaining both social and non-social ASD-related symptoms such as rigidity, repetitive behaviors and theory of mind deficits (see Hill, 2004a for review). However, EF deficits in individuals with ASD are not universal (Ozonoff and Strayer, 2001; Yerys et al., 2007) and over time it became clear that the data did not support the primary deficit hypothesis. Although we are looking at early manifestations of EF differences in ASD, we are not proposing that EF differences fully explain all ASD symptoms but instead suggest that they may contribute to some of the deficits observed in individuals with ASD.

Although the A-not-B is one of the most widely used and well-studied measures of EF in infants and young toddlers, it has important limitations. The A-not-B, used to measure EF, is not highly specific and does not simply measure one type of EF. It should also be noted that although working memory and response inhibition were discussed as being separate based on the type of trial administered, these variables are interrelated. Moreover, there are no published norms on the A-not-B and therefore the extent of EF dysfunction compared to same-aged peers is not clear. ASD diagnosis at 24-months demonstrates high classification stability over time among both clinically ascertained samples and HR sibling cohorts (Chawarska et al., 2007; Ozonoff et al., 2011; Rozga et al., 2011; Guthrie et al., 2013). However, there are potential limitations to using 24-month diagnosis or classification. In a recent study, 41% of HR siblings not initially diagnosed at 24-months were later diagnosed at 36 months (Ozonoff et al., 2015). Thus, our finding that the HR-Negative and HR-ASD groups did not differ on working memory and response inhibition could be due to some children in the HR-Negative group who may later meet diagnostic criteria for ASD. Longitudinal follow-up of these children is currently underway to assess whether changes in ASD outcomes occur over time and whether these changes reveal different patterns of early precursors to ASD.

Interestingly, our LR sample included 3 children (3.9%) who developed ASD. This rate is higher than the CDC prevalence estimate of 1.5%. Our study does not provide direct evidence for why this may have occurred. It is possible that there is a higher rate of false positive ASD diagnoses among the LR sample at 24 months. Alternatively, among our LR sample, parents with developmental concerns about other children in the family may be over represented despite screening out families with autism-related developmental concerns in older siblings. However, random sampling variability may also explain the prevalence rate of ASD in our LR sample. Since 1.5% is the point estimate in the general population, a rate of 3.9% would not likely be outside the bounds of what would be expected in random samples taken from the general population.

Studies assessing EF using a broader range of EF measures are needed (Bernier et al., 2010). This study only assessed EF twice, at 12 and 24 months. It appears that the deficits emerged during the time period that was not assessed. Future studies are needed to assess the trajectory of EFs more densely over the second year of life. Further research on EF at 12 months as related to motor development and other functional outcomes such as adaptive functioning, temperament, and emotional regulation could help clarify potential downstream effects of early EF differences in HR infants. In addition, the relationship between 24-month performance on the A-not-B and later development is not measured in this study. Longitudinal follow up through preschool and early school age in infants at risk for autism is needed to elucidate the developmental sequelae of these early group differences. Finally, studies linking early brain and EF differences would elucidate the neurobiological underpinnings of executive dysfunction in ASD.

This study underscores the importance of addressing developmental phenomenon among unaffected high-risk siblings in relation to those who develop ASD. It also highlights the importance of studying EF and motor developmental processes early and over time. Finally, if the findings in this study are replicated, stronger evidence may exist for considering both EF and motor-based interventions for affected and unaffected high-risk siblings.

## IBIS network

The Infant Brain Imaging Study (IBIS) Network is an NIH funded Autism Center of Excellence project and consists of a consortium of 8 universities in the U.S. and Canada. Clinical Sites: University of North Carolina: J. Piven (IBIS Network PI), H.C. Hazlett, C. Chappell; University of Washington: S. Dager, A. Estes, D. Shaw; Washington University: K. Botteron, R. McKinstry, J. Constantino, J. Pruett; Children's Hospital of Philadelphia: R. Schultz, S. Paterson; University of Alberta: L. Zwaigenbaum; University of Minnesota: J. Elison; Data Coordinating Center: Montreal Neurological Institute: A.C. Evans, D.L. Collins, G.B. Pike, V. Fonov, P. Kostopoulos; S. Das; Image Processing Core: University of Utah: G. Gerig; University of North Carolina: M. Styner; Statistical Analysis Core: University of North Carolina: H. Gu

## Author contributions

TJ drafted and revised the manuscript and participated in acquisition, analysis, and interpretation of the data. AE participated in manuscript revision, data interpretation, and study conception and design. SD participated in manuscript revision, data interpretation, and study conception and design. PK participated in manuscript revision and data interpretation. JW participated in manuscript revision and data interpretation. JP participated in manuscript revision and data acquisition. JE participated in manuscript revision and data interpretation. SP participated in manuscript revision and data acquisition. RS participated in manuscript revision, data interpretation, and study conception and design. KB participated in manuscript revision and study conception and design. HH participated in manuscript revision and study conception and design. JP participated in manuscript revision, data interpretation, and study conception and design.

## Funding

This work was supported by an NIH Autism Center of Excellence grant (NIMH and NICHD #HD055741 and HD055741), NIH Intellectual and Developmental Disabilities Research Center grant (#U54HD083091), the Simons Foundation (SFARI Grant 140209), and Autism Speaks (6020).

### Conflict of interest statement

The authors declare that the research was conducted in the absence of any commercial or financial relationships that could be construed as a potential conflict of interest.

## Footnotes

^1^Centers for Disease Control Autism and Developmental Disabilities Monitoring Network (2016). Available online at: http://www.cdc.gov/ncbddd/autism/addm.html
